# Plasmonic Field-Enhanced Raman Sensing Enables Rapid Trace Methanol Detection in Transformer Oil

**DOI:** 10.3390/s26165291

**Published:** 2026-08-21

**Authors:** Xiaoqin Zhang, Hongbin Zhu, Hao Liu, Jin Cao, Han Shi, Shanyuan Niu

**Affiliations:** 1 Electric Power Research Institute of State Grid Jiangsu Electric Power Co., Ltd., Nanjing 211103, China; 2 College of Engineering and Applied Sciences, Nanjing University, Nanjing 210026, China; haoliu@smail.nju.edu.cn (H.L.); 502025340017@smail.nju.edu.cn (J.C.); 602025340064@smail.nju.edu.cn (H.S.)

**Keywords:** transformer oil, methanol detection, surface-enhanced Raman spectroscopy, plasmonic field enhancement, oil-paper insulation aging

## Abstract

Methanol is a critical molecular marker for the early aging of oil-paper insulation, and its rapid detection is highly valuable for insulation condition assessment and the fault warning of power transformers. Widely used chromatographic methods require sophisticated pretreatment workflow and are not suitable for in situ monitoring. Non-destructive spectroscopic methods remain challenging due to the intrinsically small cross section of trace molecules in complex liquid environments. The rapid, direct detection of trace methanol in an oil mixture has yet to be demonstrated. In this study, a high-performance Raman-enhancing substrate was developed through hierarchical microstructure regulation, combining microscale light-trapping structures and nanoscale field-confinement sites to sense the weak Raman response of methanol in transformer oil. Direct detection of ppm-level methanol in the oil matrix was achieved, without additional adsorption enrichment or other complicated pretreatment procedures. The characteristic Raman band of methanol in transformer oil was identified, and a quantitative sensing method was established. Furthermore, the intrinsic temperature-dependent Raman response of methanol was investigated to evaluate the stability of its characteristic fingerprint bands over a broad temperature range. This work demonstrates a rapid, sensitive, and pretreatment-free spectroscopic strategy for trace methanol detection in complex oil matrices, and also sheds light on the high-sensitivity detection of small molecular markers in complex liquid environments.

## 1. Introduction

Power transformers are key assets in modern power grids, and their reliable operation is closely associated with effective fault diagnosis and condition monitoring [1]. During long-term service, oil-paper insulation systems are subjected to thermal, oxidative, hydrolytic, and electrical stresses, which can induce cellulose chain scission, decrease the degree of polymerization, weaken mechanical strength, and reduce dielectric reliability [2]. Since the direct sampling of solid insulation from operating transformers is destructive and impractical, aging-related components dissolved in transformer oil have become important non-invasive indicators for insulation diagnosis and real-time condition monitoring [3,4]. Among these components, methanol has attracted increasing attention as a sensitive marker for early oil-paper insulation aging. It is associated with the rupture of 1,4-β-glycosidic bonds in cellulose and can be generated during the early depolymerization of insulating paper [5]. Previous aging experiments and field studies have shown that methanol concentration in transformer oil is correlated with the degradation state of paper insulation, including the decrease in degree of polymerization and deterioration of mechanical properties [6,7]. Therefore, rapid methanol detection is highly valuable for the early warning and life assessment of transformer insulation systems [8,9].

Chromatographic techniques are currently the most widely used methods for methanol analysis in transformer oil. Static headspace gas chromatography coupled with mass spectrometry has been used to quantify methanol and ethanol generated from cellulose degradation in mineral insulating oils, showing high sensitivity and good separation capability [10]. Gas chromatography with suitable detectors can also provide reliable quantitative results for alcohol markers in complex oil matrices [11]. In addition, headspace extraction and microextraction strategies have been introduced to improve the detection of volatile alcohols before chromatographic analysis [12]. These methods are accurate, selective, and mature, but they usually require specialized instruments, sample transportation, equilibrium or extraction procedures, and relatively long analysis cycles. Therefore, they are less suitable for on-site rapid screening, quasi-in situ analysis, or the real-time monitoring of transformer oil [13].

Raman spectroscopy has been explored for methanol analysis in transformer oil because it can directly obtain molecular fingerprint information from liquid samples [14]. Gu et al. investigated laser Raman spectroscopy using a confocal liquid-detection platform and selected the methanol band near 1030 cm^−1^ for quantitative analysis; however, extraction was further introduced to improve sensitivity, indicating that the direct Raman response of methanol in oil remained insufficient [15]. To enhance spectral detectability, Zhang et al. reported a SERS-related method in which methanol was first extracted from transformer oil, oxidized, and reacted with chromotropic acid to form a colored derivative before spectral detection [16]. Zhang et al. also performed theoretical calculations to clarify the influence of intermolecular interactions and electric fields on the Raman/SERS response of methanol in transformer oil [17]. Chen et al. developed a derivatization reaction-based SERS method using Ag/ZnO composite nanoflower substrates, improving methanol detection through chemical conversion and enhanced Raman readout [18]. These studies demonstrate the feasibility of Raman/SERS-based methanol analysis. However, related methanol detection strategies for transformer oil commonly involve extraction, oxidation, derivatization, or other pretreatment procedures, and the direct rapid detection of trace methanol in complex transformer oil without complicated pretreatment remains insufficiently demonstrated [19,20,21]. Recent studies on transformer condition assessment also highlight the importance of reliable feature extraction and rapid diagnostic information from oil-related state quantities [22].

In this work, we report a plasmonic field-enhanced Raman sensing strategy for rapid trace methanol detection in transformer oil. A high-performance Raman-enhancing substrate was constructed through micro/nanostructural regulation and localized electromagnetic field engineering, enabling significant amplification of the weak Raman response of methanol in the oil matrix. Benefiting from the enhanced local plasmonic field, the direct detection of trace methanol in oil was achieved within 60 s (spectral acquisition time: 30 s) without additional adsorption enrichment, oil–gas separation, chemical extraction, or other complicated pretreatment procedures. The characteristic Raman band of methanol in transformer oil was identified, and a concentration-dependent quantitative detection method was established. Furthermore, the temperature-dependent Raman response of methanol was investigated by analyzing temperature-induced peak-position variations, providing a basis for spectral recognition and quantitative correction under practical operating conditions. This study demonstrates a rapid, sensitive, and pretreatment-free spectroscopic route for trace methanol detection in complex oil matrices, offering a potential route for non-destructive screening and condition assessment of transformer oil-paper insulation systems.

## 2. Materials and Methods

### 2.1. Reagents and Instrumentation

Methanol (analytical grade; Shanghai Aladdin Biochemical Technology Co., Ltd., Shanghai, China) was used as the target analyte for transformer oil aging marker detection. A commercially available GTL-derived electrical insulating oil (Shell Diala S4 ZX-I; Shell plc, London, UK), provided by the Electric Power Research Institute of State Grid Jiangsu Electric Power Co., Ltd. (Nanjing, China), was used as the transformer-oil matrix. According to the manufacturer’s technical data sheet, the oil has a typical density of 805 kg m^−3^ at 20 °C, a kinematic viscosity of 9.8 mm^2^ s^−1^ at 40 °C, a flash point of 191 °C, and a pour point of −45 °C. The oil was used as received and stored in a sealed container before sample preparation. Standard methanol oil samples with concentrations of 10, 50, 100, 200, and 400 μL/L were prepared for concentration-dependent Raman measurements. Methanol-containing oil samples with relatively high concentrations, including 1000 μL/L and 100 mL/L, were prepared for characteristic peak identification in the oil matrix. Before testing, all oil samples were thoroughly mixed to ensure sample homogeneity.

Acetone and isopropanol (analytical reagent grade; Shanghai Aladdin Biochemical Technology Co., Ltd., Shanghai, China), and ultrapure water were used for substrate cleaning, and high-purity nitrogen was used to dry the substrates before Raman measurements. Rhodamine 6G (R6G; analytical reagent grade; Shanghai Aladdin Biochemical Technology Co., Ltd., Shanghai, China) was used as a probe molecule to evaluate the Raman enhancement performance of the prepared substrates.

Raman spectra were collected using a portable fiber-optic Raman system consisting of a QE Pro spectrometer (Ocean Optics, Orlando, FL, USA), a 638 nm excitation laser, and a fiber-optic Raman probe (Ocean Optics, Orlando, FL, USA). Spectral acquisition and instrument control were performed using OceanView 2.0 software. The spectral resolution was approximately 10 cm^−1^, and the laser power measured at the sample was 30 mW. No polarization-maintaining fiber, polarizer, or analyzer was used; therefore, the polarization state at the sample was not precisely controlled. Nevertheless, the Raman probe was fixed relative to the substrate, and the optical configuration and acquisition conditions were kept consistent throughout all measurements. Unless otherwise specified, the integration time was 10 s, and three accumulations were collected for each spectrum. The effective sampling region corresponded to the laser-illuminated area on the Raman-enhancing substrate.

### 2.2. Fabrication and Characterization of Raman-Enhancing Substrates

A hierarchical Au-coated silicon Raman-enhancing substrate was fabricated by photolithography, ICP etching, Ag-assisted metal-assisted chemical etching (MACE), and Au deposition. Briefly, AZ5214E photoresist was spin-coated on an n-type Si (100) wafer at 4000 rpm for 30 s, soft-baked at 90 °C for 90 s, exposed at 120 mJ cm^−2^, and developed in AZ 726 MIF for 60 s. Microscale grooves were formed using a Bosch ICP process with SF_6_/C_4_F_8_ plasma at 600 W, 10 mTorr, and a bias power of 11 W for 14 cycles. Ag nanoparticles were deposited in a solution containing 3.0 M HF and 1.5 mM AgNO_3_ for 60 s, followed by MACE in 5.0 M HF and 0.4 M H_2_O_2_ for 5 min at room temperature. After removal of the residual Ag using HNO_3_, a 50 nm Au layer was deposited by electron-beam evaporation at 0.5 Å s^−1^. The substrate morphology and elemental distribution were characterized by optical microscopy, SEM, and EDS, and its absorption, reflection, and transmission spectra were measured over 400–1250 nm. The Raman enhancement performance was evaluated using R6G solutions of different concentrations under identical acquisition conditions, with detectable characteristic signals obtained down to 10^−13^ M.

### 2.3. Raman Spectral Acquisition of Methanol and Methanol-Containing Oil Samples

A concentrated aqueous methanol solution containing 80% (*v*/*v*) methanol was used as the reference sample for Raman-band identification. The aqueous solution was selected to reduce spectral fluctuations caused by the rapid evaporation of methanol. A small volume of sample was placed on a clean sample holder, and Raman spectra were acquired using a QE Pro Raman spectrometer with 638 nm excitation, 10 s integration time, and three accumulations.

For oil-phase detection, blank transformer oil and methanol-containing transformer oil samples were tested under identical conditions. The blank oil spectrum was collected as the background reference. Methanol-containing oil samples were directly dropped onto the Raman-enhancing substrate surface and measured with the fiber-optic Raman probe. No adsorption enrichment, oil–gas separation, chemical extraction, or other pretreatment procedures were applied before spectral acquisition.

For concentration-dependent measurements, standard methanol oil samples with concentrations of 10, 50, 100, 200, and 400 μL/L were tested using the same substrate type and Raman acquisition parameters. For each concentration, spectra were collected under identical optical and acquisition conditions to ensure comparability among different samples.

### 2.4. Calibration Curve Construction and Quantitative Analysis

The collected Raman spectra were processed for the quantitative analysis of methanol in transformer oil. The Raman band near 1034 cm^−1^ was selected as the characteristic analytical band of methanol in the oil matrix. For each concentration, the local spectral region from 1020 to 1050 cm^−1^ was extracted for further analysis. To reduce the influence of oil background and local baseline fluctuation, baseline correction was first performed within this spectral window.

After baseline correction, Gaussian peak fitting was applied to resolve the methanol-related Raman band near 1034 cm^−1^. The fitted peak area of the 1034 cm^−1^ band was calculated and used as the quantitative Raman response. A calibration curve was then constructed by plotting the fitted peak area against methanol concentration in the range of 10–400 μL/L. Linear regression was performed to establish the quantitative model, and the coefficient of determination was used to evaluate the linear fitting performance.

Baseline correction, Gaussian peak fitting, linear regression, statistical analysis, and figure preparation were performed using MATLAB R2025b (The MathWorks, Inc., Natick, MA, USA).

### 2.5. Temperature-Dependent Raman Measurements

Temperature-dependent Raman measurements were conducted from −80 to 50 °C to investigate the spectral response of methanol under different temperature conditions. Methanol samples were tested at selected temperatures using the same Raman spectrometer and acquisition parameters. For each temperature point, the Raman spectrum was acquired with 638 nm excitation, 10 s integration time, and three accumulations.

The Raman peaks near 1034 and 1453 cm^−1^ were selected for temperature-dependent peak position analysis. Peak positions were extracted from the measured spectra under different temperature conditions. The obtained results were used to evaluate the intrinsic temperature stability of the characteristic methanol Raman bands.

## 3. Results and Discussion

### 3.1. Structural Characterization and Raman Enhancement Performance of the Substrate

**Figure 1:**
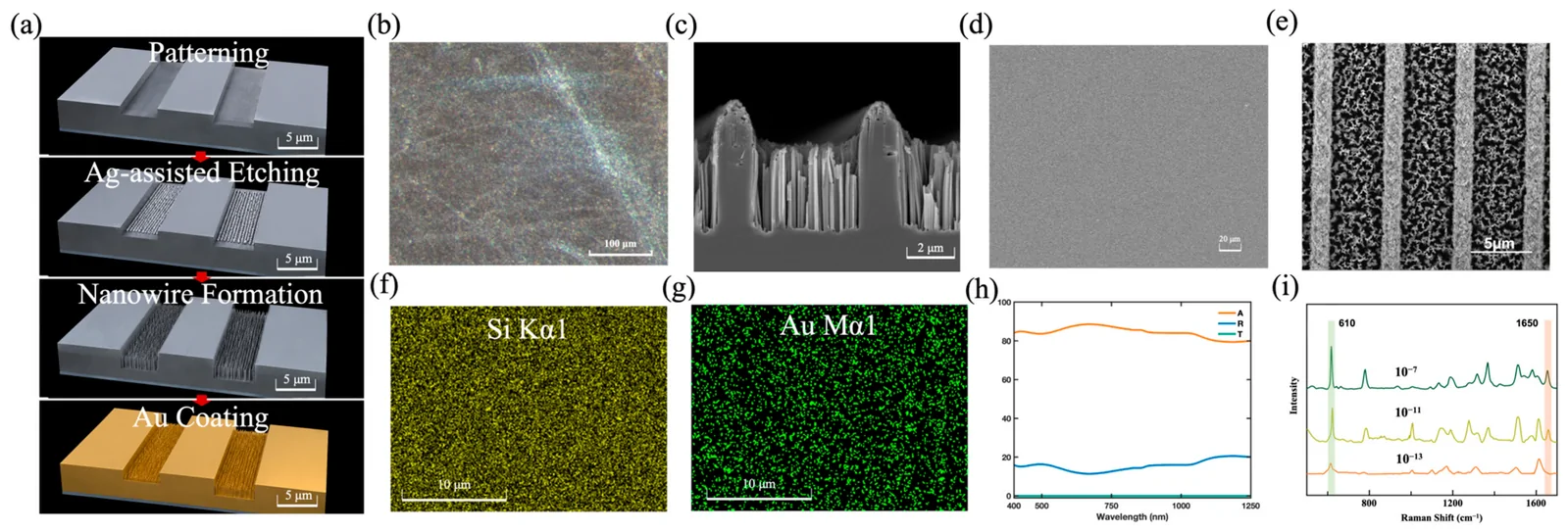
(**a**) Schematic illustration of the fabrication process. (**b**) Optical microscopy image of the fabricated substrate. (**c**) Cross-sectional SEM image of the hierarchical microgroove–nanowire structure. (**d**) Low-magnification top-view SEM image showing the large-area morphology and structural uniformity of the substrate. (**e**) High-magnification top-view SEM image revealing the nanoscale surface morphology. (**f**,**g**) EDS elemental mappings of Si Kα and Au Mα. (**h**) Optical absorption, reflection, and transmission spectra of the substrate over the wavelength range of 400–1250 nm. (**i**) Raman spectra of the R6G solutions with different concentrations measured on the substrate.

The hierarchical Au-coated silicon Raman-enhancing substrate was fabricated through photolithography, ICP etching, Ag-assisted metal-assisted chemical etching, and subsequent Au deposition, as described in Section 2.2 and schematically illustrated in Figure 1a. Ag nanoparticles served as temporary catalysts for silicon nanowire formation and were removed after MACE, whereas the subsequently deposited Au layer constituted the plasmonically active component of the final substrate.

The optical microscopy image in Figure 1b shows a macroscopically continuous and uniform patterned surface. The cross-sectional SEM image in Figure 1c clearly reveals the hierarchical architecture, in which dense silicon nanowire arrays are distributed within the microscale grooves. The low-magnification top-view SEM image in Figure 1d further demonstrates the large-area uniformity of the fabricated surface, while the high-magnification image in Figure 1e reveals a rough nanoscale morphology with abundant interconnected features.

The EDS elemental mappings in Figure 1f,g show the distributions of Si and Au, respectively. The Si signal confirms the underlying hierarchical silicon framework, while the broadly distributed Au signal verifies the successful deposition of Au on the final substrate. These results clarify that the plasmonic activity of the sensing surface originates from the deposited Au coating.

Based on the hierarchical morphology observed in this study and the enhancement mechanisms reported for related plasmonic substrates, the observed Raman enhancement is likely associated with the combined effects of microscale light trapping and nanoscale plasmonic-field concentration. The microscale patterned features may promote multiple reflection and scattering, while the Au-coated silicon nanowires, interwire gaps, and junctions are expected to facilitate localized surface plasmon excitation and electromagnetic-field enhancement. Consistent with this interpretation, the characteristic Raman signals of R6G remained distinguishable at concentrations down to 10^−13^ mol/L, demonstrating the strong Raman-enhancement performance of the substrate. Nevertheless, the relative contributions of these enhancement effects remain to be further investigated.

The optical absorptance, reflectance, and transmittance spectra of the substrate over 400–1250 nm are presented in Figure 1h. The substrate exhibited broad optical absorptance and nearly negligible transmittance across the measured wavelength range, indicating strong interaction between incident light and the hierarchical surface. This broadband optical response is consistent with the combined effects of light trapping by the microscale grooves and plasmonic coupling associated with the Au-coated nanostructures.

The Raman enhancement performance was further evaluated using R6G as a probe molecule. As shown in Figure 1i, the characteristic R6G bands, including those near 610 and 1650 cm^−1^, remained distinguishable at the lowest tested concentration of 10^−13^ M. This result demonstrates the strong Raman enhancement capability of the hierarchical Au-coated substrate and supports its subsequent application to weak methanol-signal detection in transformer oil.

### 3.2. Identification of Methanol Raman Signatures in Transformer Oil

**Figure 2:**
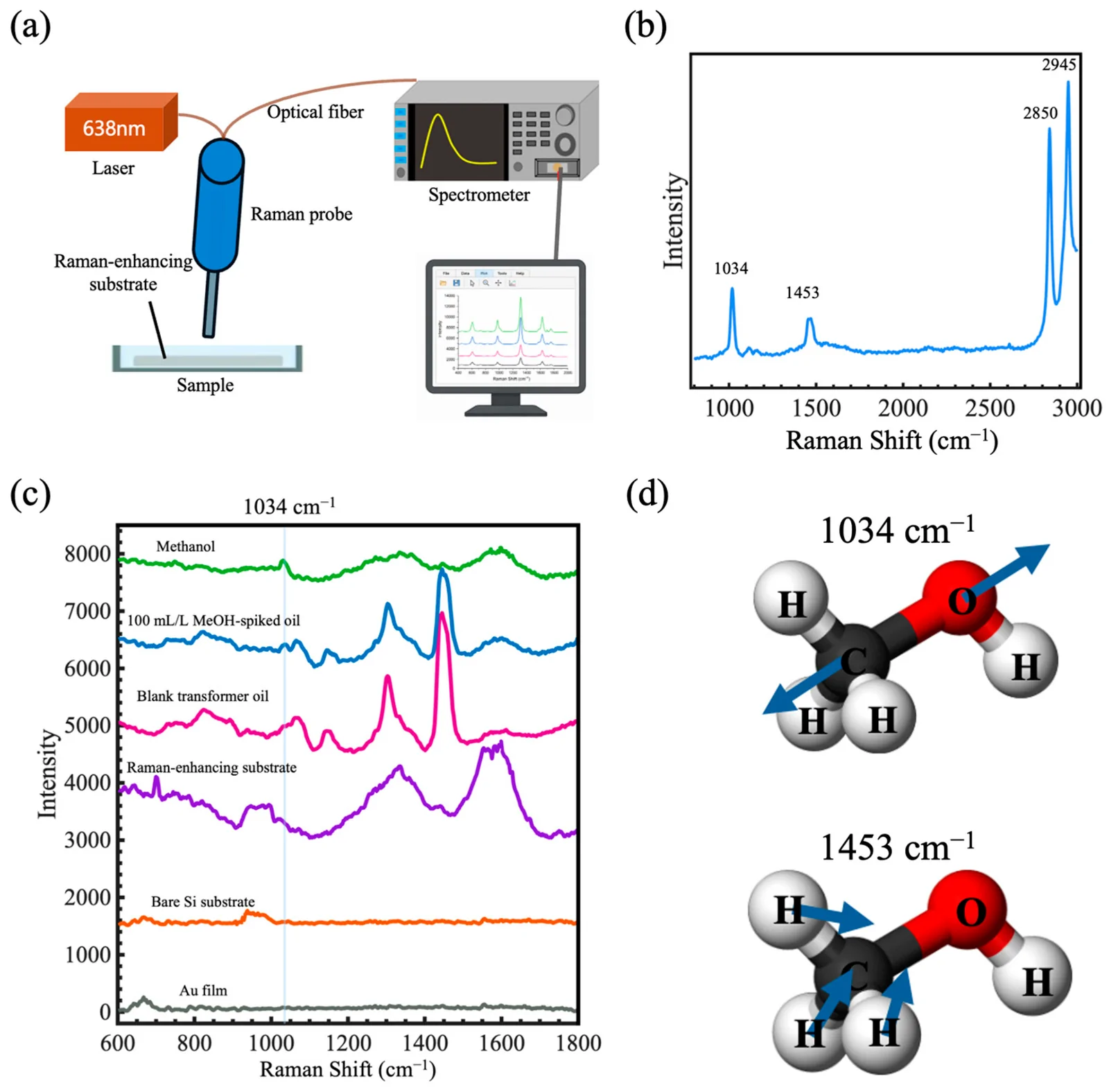
(**a**) Schematic illustration of Raman measurement system. (**b**) Raman spectrum of the 80% (*v*/*v*) aqueous methanol reference solution, showing characteristic bands near 1034, 1453, 2850, and 2945 cm^−1^. (**c**) Raman spectra of the 80% (*v*/*v*) aqueous methanol reference solution, a high-concentration methanol-spiked transformer-oil sample (100 mL L^−1^) used solely for qualitative band identification, blank transformer oil, the Au-coated hierarchical Si substrate, bare Si, and an Au film. (**d**) Schematic representations of the molecular vibration modes associated with the methanol Raman bands at 1034 and 1453 cm^−1^.

The Raman measurement system used in this work is schematically illustrated in Figure 2a. The 638 nm excitation laser was coupled to the Raman probe through an optical fiber, and the Raman signals were collected by a fiber optic spectrometer and transmitted to a computer.

The 80% (*v*/*v*) aqueous methanol reference solution was first measured to identify the characteristic Raman bands used for subsequent oil-phase analysis. As shown in Figure 2b, the Raman spectrum of methanol exhibited several characteristic bands located at approximately 1034, 1453, 2850, and 2945 cm^−1^. Among these bands, the peak near 1034 cm^−1^ is assigned to the C-O stretching vibration of methanol, while the peak near 1453 cm^−1^ is mainly associated with the deformation or bending vibration of the CH_3_ group. The high-wavenumber bands at approximately 2850 and 2945 cm^−1^ originate from C-H stretching vibrations. Together, these characteristic Raman bands provide the molecular fingerprint information of methanol.

To further clarify the vibrational origin of the two main low-wavenumber peaks, schematic vibration modes were introduced, as shown in Figure 2d. For the 1034 cm^−1^ band, the dominant vibration is the stretching motion along the C-O bond, in which the carbon and oxygen sides move relative to each other, leading to periodic elongation and contraction of the C-O bond. In contrast, the 1453 cm^−1^ band corresponds mainly to CH_3_ deformation/bending vibration. In this mode, the hydrogen atoms in the methyl group swing around the carbon atom, causing periodic changes in the C-H bond angles rather than simple bond-length stretching. This vibrational-mode assignment confirms that the 1034 cm^−1^ band is a chemically specific fingerprint of the C-O structure in methanol, whereas the 1453 cm^−1^ band reflects the methyl-group deformation.

For transformer oil analysis, the C-O stretching band near 1034 cm^−1^ is particularly important. Although methanol exhibits strong Raman responses in the high-wavenumber C-H stretching region, transformer oil contains abundant hydrocarbon structures that also generate intense C-H vibration bands in a similar spectral region. Therefore, direct use of the high-wavenumber C-H stretching bands for methanol identification would suffer from strong matrix interference. The 1453 cm^−1^ band is also easily affected by the hydrocarbon background of transformer oil because it is related to methyl or methylene deformation vibrations that are widely present in oil molecules. In comparison, the C-O stretching peak near 1034 cm^−1^ is more characteristic of methanol and is less affected by the dominant hydrocarbon vibrations of transformer oil. Therefore, this band was selected as the primary analytical peak for methanol recognition and subsequent quantitative analysis.

After the intrinsic Raman features of methanol were identified, methanol-containing transformer oil samples were measured to verify whether the methanol fingerprint could still be recognized in the complex oil matrix. As shown in Figure 2c, the comparison among methanol, MeOH-spiked transformer oil, blank transformer oil, the Au-coated hierarchical Si Raman-enhancing substrate, bare Si substrate, and Au film demonstrated that the methanol-related spectral response appeared near 1034 cm^−1^ in the transformer oil matrix. No corresponding feature was observed at this position in the spectra of the blank transformer oil, Raman-enhancing substrate, bare Si substrate, or Au film, excluding an appreciable contribution from the oil matrix or substrate materials. This peak position is consistent with the 1034 cm^−1^ C-O stretching band of methanol, indicating that the characteristic methanol vibration is preserved after methanol is dispersed in the transformer oil matrix.

The oil-phase identification result confirms that the C-O stretching vibration of methanol is preserved and spectroscopically recognizable in transformer oil. More importantly, the oil samples were directly measured on the Raman-enhancing substrate without adsorption enrichment, oil–gas separation, chemical extraction, oxidation, or derivatization. Therefore, the observation of the 1034 cm^−1^ band in methanol-containing oil provides direct evidence that the weak methanol fingerprint can be accessed from the oil matrix through substrate-enabled plasmonic enhancement. This result establishes the spectral basis for the following concentration-dependent detection and quantitative calibration.

### 3.3. Concentration-Dependent Response and Quantitative Detection of Methanol in Oil

After determining the characteristic methanol peak in the oil matrix, standard methanol-containing transformer oil samples with concentrations of 10, 50, 100, 200, and 400 μL/L were measured to evaluate the quantitative capability of the proposed Raman sensing method. Each concentration was independently measured three times under identical experimental conditions. Since the methanol-related response in oil is relatively weak and is easily affected by the complex hydrocarbon background, the spectral region from 1020 to 1050 cm^−1^ was selected for local analysis around the methanol characteristic band.

**Figure 3:**
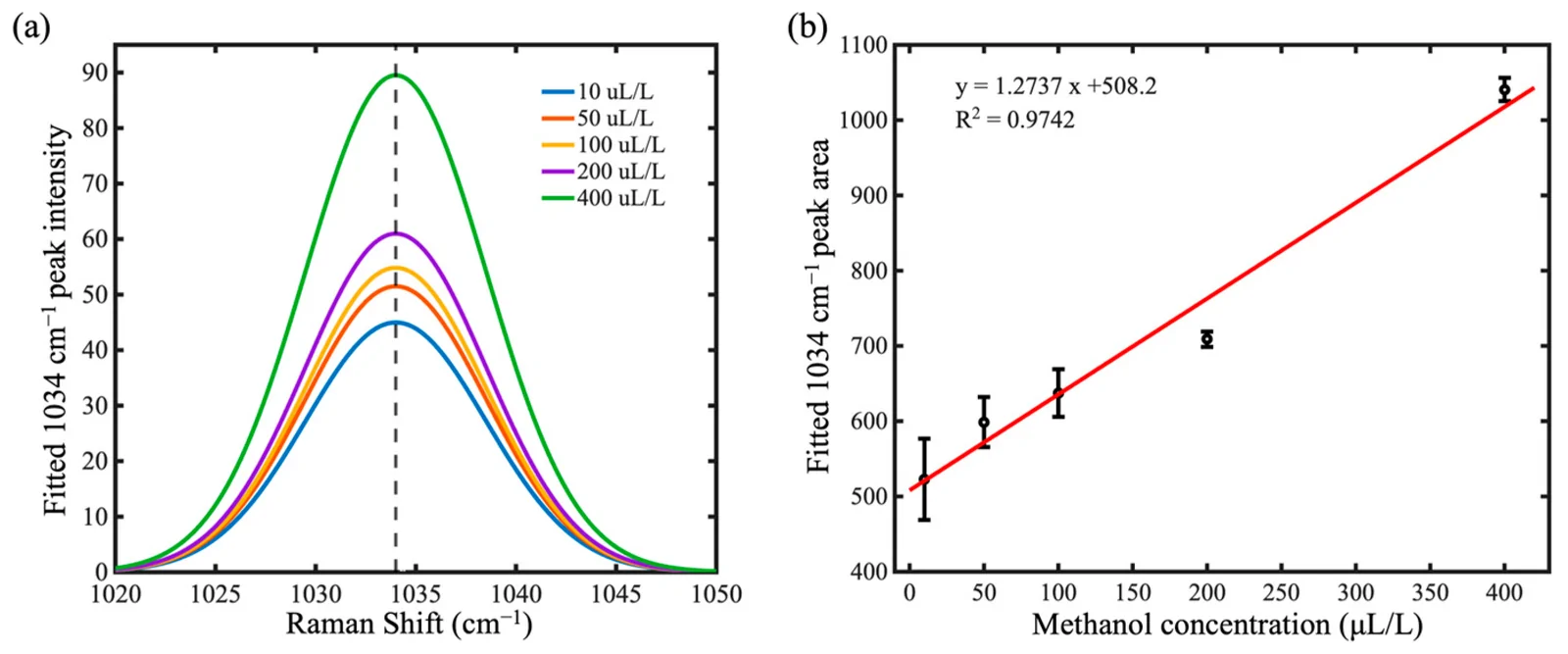
(**a**) Baseline-corrected Raman spectra of methanol-containing transformer oil samples at concentrations ranging from 10 to 400 μL/L in the spectral region of 1020–1050 cm^−1^. The methanol characteristic band near 1034 cm^−1^ was extracted by Gaussian peak fitting. (**b**) Calibration curve constructed using the fitted peak area of the 1034 cm^−1^ band, showing the linear relationship between methanol concentration and Raman response. Each concentration was measured independently multiple times, and the error bars represent the standard deviation of three replicate measurements.

For each concentration, baseline correction was first performed within the 1020–1050 cm^−1^ region to suppress the influence of the local oil background and baseline fluctuation. After baseline removal, Gaussian peak fitting was applied to extract the methanol-related spectral component near 1034 cm^−1^. As shown in Figure 3a, the fitted 1034 cm^−1^ peak became progressively stronger with increasing methanol concentration, confirming that the enhanced Raman response of methanol retains clear concentration-dependent behavior in the transformer oil matrix.

Compared with directly using raw peak height, Gaussian fitting provides a more reliable way to quantify the weak methanol signal in a complex oil background [23]. The fitting process resolves the characteristic methanol contribution from the local spectral window and reduces the influence of noise, baseline variation, and nearby matrix interference. Therefore, the fitted peak area of the 1034 cm^−1^ band was selected as the quantitative parameter for calibration. For each concentration, the mean fitted peak area obtained from three replicate measurements was used to construct the calibration curve.

Based on the fitted peak areas, a concentration-dependent Raman response was obtained over the tested range of 10–400 μL/L. As shown in Figure 3b, the fitted peak area exhibited an overall linear relationship with methanol concentration, with a coefficient of determination of 0.9742. The deviations observed at 50 and 200 μL/L may be attributed to the spatial heterogeneity of plasmonic hotspots on the substrate, variations in the local distribution of the oil sample, and uncertainty in extracting the relatively weak 1034 cm^−1^ peak from the complex oil background. The error bars obtained from multiple measurements reflect these experimental variations. Despite these variations, the fitted Raman response exhibited a preliminary concentration-dependent relationship with methanol concentration under fixed experimental conditions. More importantly, the measurement was performed directly in the oil matrix without adsorption enrichment, oil–gas separation, chemical extraction, oxidation, or derivatization, highlighting the potential of this method for the rapid analysis of transformer-oil samples and its possible future development toward on-site applications. To place the proposed method in the context of existing analytical techniques, representative methods for methanol detection in transformer oil are compared in Table 1, with a particular emphasis on sample pretreatment and analytical range.

As summarized in Table 1, chromatographic methods provide high analytical sensitivity but generally involve headspace equilibration, internal-standard addition, gas–liquid phase transfer, and chromatographic separation. Previously reported spectroscopic and SERS-based methods reduce the dependence on conventional chromatography but commonly require extraction, oxidation, chemical derivatization, or phase transfer to improve methanol detectability. In contrast, the present method directly analyzes methanol in the original transformer-oil matrix without any sample pretreatment. Although some established methods provide lower detection limits, the principal advantage of the proposed approach lies in its simplified workflow and direct access to the methanol Raman fingerprint in the complex oil matrix.

From an application perspective, the concentration-dependent response of the 1034 cm^−1^ band is meaningful for transformer condition monitoring. Methanol is closely associated with early cellulose depolymerization, and the rapid quantitative detection of low-level methanol in transformer oil can provide valuable diagnostic information before severe insulation degradation occurs. The principal contribution of this work is the demonstration that the Raman response of a trace small-molecule aging marker can be directly identified and evaluated in the tested insulating-oil matrix using a hierarchical Raman-enhancing substrate.

### 3.4. Temperature-Dependent Raman Stability of Methanol

The temperature-dependent experiment in this section was performed using methanol to evaluate the robustness of Raman fingerprint recognition under variable thermal conditions. Since transformer-oil temperature varies with load, ambient conditions, and thermal history, it is important to determine whether the characteristic methanol Raman bands remain distinguishable over a broad temperature range. Raman spectra were collected from −80 to 50 °C, with particular attention to the normal operating-temperature range of approximately −20 to 50 °C. The representative bands near 1034 and 1453 cm^−1^ were selected to evaluate temperature-induced spectral variations.

**Figure 4:**
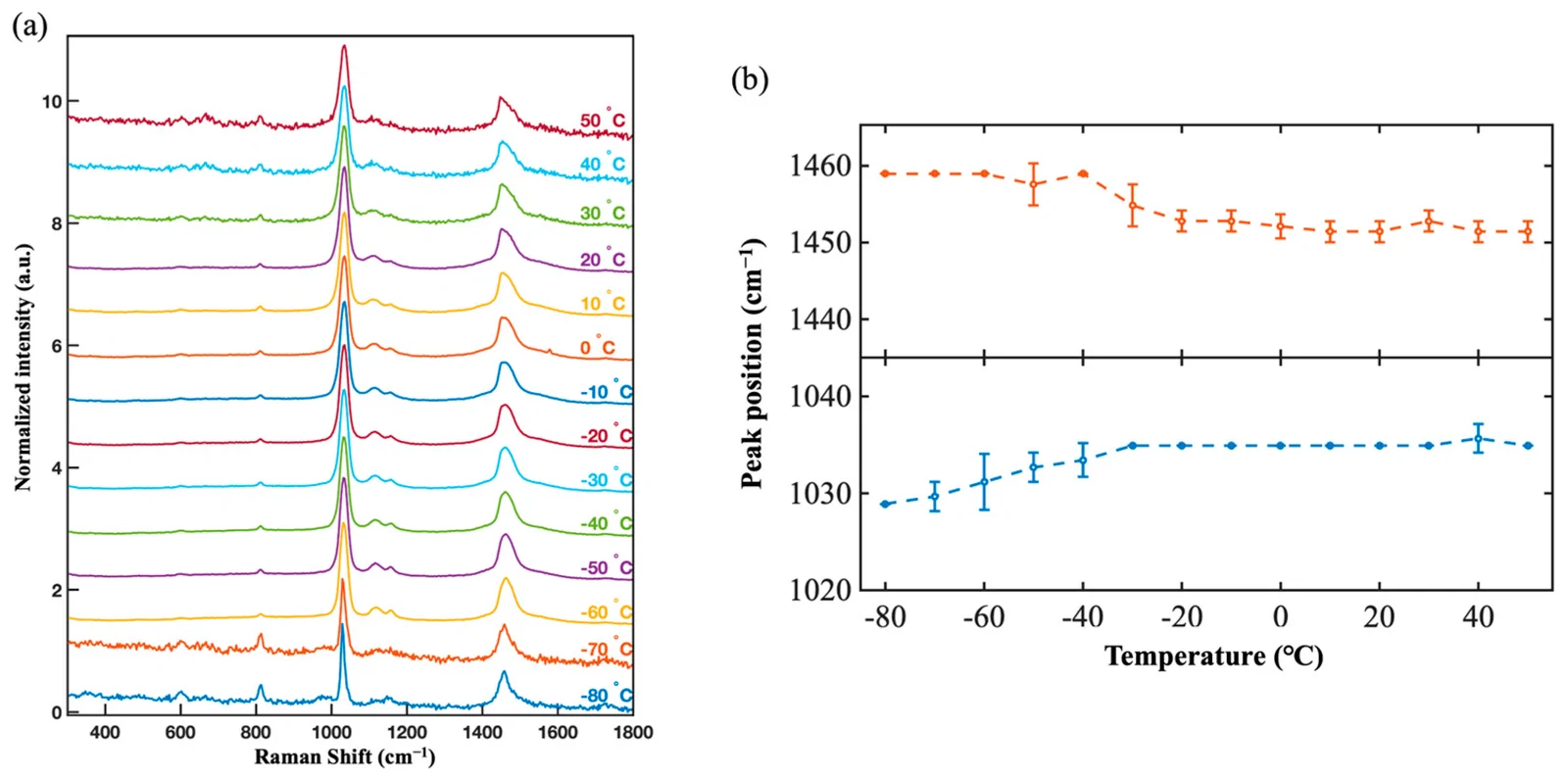
(**a**) Raman spectra of methanol collected at different temperatures from −80 to 50 °C. (**b**) Peak-position variations of the characteristic methanol bands near 1034 and 1453 cm^−1^ as a function of temperature. Error bars represent the standard deviation from four parallel measurements.

As shown in Figure 4a, the main Raman fingerprints of methanol remained clearly observable throughout the tested temperature range. With increasing temperature, no new Raman bands appeared, and no characteristic peaks disappeared or split, indicating that temperature variation did not change the fundamental vibrational identity of methanol. The two selected bands exhibited only limited peak-position variations, suggesting that the methanol Raman fingerprints are thermally stable and can still be reliably recognized under variable temperature conditions.

More specifically, the C-O stretching band near 1034 cm^−1^ showed a slight blue shift as the temperature increased. Its peak position gradually shifted from approximately 1029 cm^−1^ at low temperature to around 1035 cm^−1^ at higher temperature, and then remained nearly stable in the higher-temperature region. This behavior may be related to temperature-dependent changes in intermolecular interactions and the local hydrogen-bonding environments of methanol. In contrast, the CH_3_ deformation band near 1453 cm^−1^ exhibited a slight red shift with increasing temperature. Its peak position decreased from approximately 1459 cm^−1^ at low temperature to around 1451 cm^−1^ at higher temperature. The opposite shift directions of these two bands indicate that different vibrational modes respond differently to thermal variation, which should be considered when using methanol Raman features for precise spectral calibration.

Although the peak positions changed slightly with temperature, the overall shifts were small and the characteristic bands remained distinguishable. This result suggests that temperature variation may introduce minor spectral deviations, but it does not compromise the feasibility of methanol identification based on the 1034 and 1453 cm^−1^ Raman bands. For practical transformer oil detection, the 1034 cm^−1^ band remains the more suitable analytical feature because it is more specific to the C-O stretching vibration of methanol and is less affected by hydrocarbon background than the 1453 cm^−1^ band. Therefore, the temperature-dependent results provide an experimental basis for subsequent temperature correction and robust quantitative modeling under practical operating conditions.

From an application perspective, the temperature-dependent measurements were performed to evaluate the robustness of methanol Raman fingerprint recognition. Within the normal operating-temperature range of approximately −20 to 50 °C, the characteristic methanol Raman peaks remained distinguishable, with limited peak-position variations. However, the influence of temperature on Raman intensity and the concentration-dependent response was not evaluated in the present study. Temperature-specific calibration curves will therefore be investigated in future work.

## 4. Conclusions

This study developed a rapid Raman sensing strategy for the concentration-dependent analysis of trace methanol in transformer oil. Plasmonic field enhancement provided by a hierarchical Au-coated silicon substrate enabled the identification of the weak Raman response of methanol in complex insulating oil matrices. The Raman-enhancing substrate was prepared through lithographic patterning, ICP etching, Ag-assisted MACE-induced nanowire formation, removal of the residual Ag catalyst, and subsequent Au deposition. Optical microscopy, cross-sectional and top-view SEM images at different magnifications, and EDS elemental mappings of Si and Au confirmed the large-area structural uniformity, hierarchical micro/nanostructure, and Au distribution of the final substrate. The optical absorptance, reflectance, and transmittance spectra over 400–1250 nm further demonstrated its broadband optical response. The combination of microscale light-trapping structures and Au-coated nanoscale field-confinement sites may contribute to the enhancement of weak Raman signals. The characteristic R6G bands remained distinguishable at the lowest tested concentration of 10^−13^ M, demonstrating the strong Raman enhancement capability of the substrate and supporting its use for trace molecular detection in liquid environments.

The Raman fingerprints of methanol were systematically identified. Methanol exhibited characteristic bands near 1034, 1453, 2850, and 2945 cm^−1^, among which the C–O stretching band near 1034 cm^−1^ was selected as the primary analytical peak. After methanol was introduced into transformer oil, this feature was confirmed near 1034 cm^−1^, indicating that the methanol fingerprint can be preserved and recognized in the oil matrix. No corresponding feature was observed at this position for blank transformer oil, the Au-coated hierarchical substrate, bare Si, or the Au film, excluding an appreciable contribution from the oil matrix or substrate materials. This result shows that methanol can be directly detected through engineered plasmonic enhancement without conversion into another derivative.

The rapid and direct detection of trace methanol was further evaluated using methanol-spiked transformer-oil samples over the concentration range of 10–400 μL L^−1^. After baseline correction and Gaussian peak fitting in the 1020–1050 cm^−1^ spectral region, the fitted area of the methanol-related band near 1034 cm^−1^ exhibited an overall concentration-dependent relationship, with a coefficient of determination of 0.9742 based on triplicate measurements. The spectral acquisition time was 30 s, and no adsorption enrichment, oil–gas separation, chemical extraction, oxidation, derivatization, or phase transfer was performed before measurement. These results support the feasibility of rapid and pretreatment-free trace-methanol detection directly in the tested transformer-oil matrix.

Temperature-dependent Raman measurements further showed that the main methanol fingerprint bands remained distinguishable from −80 to 50 °C. Only slight peak shifts were observed, with no peak formation, disappearance, or splitting. These results demonstrate the robustness of Raman-based methanol identification under variable thermal conditions.

Future work should focus on improving analytical performance and advancing practical validation. The present proof-of-concept study primarily establishes the sensitivity and feasibility of pretreatment-free Raman detection directly in transformer oil. For sensitivity enhancement, the groove geometry, nanowire density, Au-coating morphology, and hotspot coupling strength should be further optimized to strengthen local electromagnetic enhancement, improve signal uniformity, and reduce the detection limit for methanol. Surface/interface regulation may also be introduced to increase the accessibility of methanol molecules to plasmonic hotspots. To further advance this spectroscopic sensing technology toward on-site deployment, future studies should improve substrate fabrication throughput, evaluate selectivity and cross-interference from representative oil-aging products such as ethanol, acetone, and acetic acid, and validate the method using real aged transformer-oil samples and standard GC or GC-MS measurements. Large-scale data collection, standardized testing workflows, and condition-assessment models based on aged transformers will also be required. By integrating the Raman-enhancing substrate with fiber-optic probes, microfluidic sampling modules, and portable spectrometers, the method could be further developed into a compact quasi-in situ or online monitoring platform. Recent advances in fiber-optic photoacoustic spectroscopy have demonstrated compact, non-invasive, and resonance-enhanced trace-gas sensing, further highlighting the potential of fiber-optic architectures for field-deployable optical sensing platforms [24,25]. Combined with robust spectral recognition and quantitative modeling, the proposed strategy may progress from laboratory validation toward the field-oriented early warning of transformer oil-paper insulation aging. In addition, the potential interference from oil-oxidation products that also contain C–O bonds should be considered in practical applications, as they may lead to the overestimation of methanol. Future validation using real aged oils and complementary spectral deconvolution or chromatographic cross-checking will be necessary to ensure reliable quantification.

## Figures and Tables

**Table 1:** Comparison of representative methods for methanol detection in transformer oil.

Detection Method	Pretreatment Required	Pretreatment Procedure	Reported Range or Detection Limit
Static HS-GC-MS [10]	Yes	Addition of an isotopically labeled internal standard, followed by sealed-vial headspace equilibration and GC-MS analysis	Method detection limit: 4 ng g^−1^
UV–Vis spectroscopy combined with chemometric analysis [19]	Yes	Extraction from transformer oil, oxidation with KMnO_4_, and reaction with chromotropic acid	Minimum detection limit: 10 ppb
Derivatization reaction-based SERS using Ag/ZnO nanoflowers [18]	Yes	Oxidation with acidic KMnO_4_, followed by AHMT derivatization and SERS measurement	Linear range: 1–10,000 μg L^−1^; LOD: 0.87 μg L^−1^
Extraction-assisted SERS using a silver nanosubstrate [21]	Yes	Extraction of methanol from transformer oil into an aqueous phase, followed by SERS measurement using a silver nanosubstrate	Minimum detectable concentration: 1.8 mg L^−1^
Plasmonic field-enhanced Raman spectroscopy	No	Direct measurement of methanol-containing transformer oil without extraction, headspace separation, adsorption enrichment, or phase transfer	Quantitative range: 10–400 μL L^−1^

## Data Availability

The original contributions presented in this study are included in the article. Further inquiries can be directed to the corresponding author.

## Abbreviations

The following abbreviations are used in this manuscript:

SERS	Surface-enhanced Raman spectroscopy
R6G	Rhodamine 6G
SEM	Scanning electron microscopy
ICP	Inductively coupled plasma
MACE	Metal-assisted chemical etching
